# Comparison of Flexible and Navigable Suction Ureteral Access Sheath Versus Conventional Ureteric Access Sheath in RIRS: A Systematic Review and Meta-Analysis

**DOI:** 10.5152/tud.2026.25119

**Published:** 2026-04-09

**Authors:** Ankur Mittal, Anumanchi Dattasai Subramanyam, Vikas Kumar Panwar, Kunal Malhotra, Kirti Rana, Dharam Dev, Shikhar Garg

**Affiliations:** Department of Urology, All India Institute of Medical Sciences, Rishikesh, India

**Keywords:** Control, equipment design, instrumentation, kidney calculi, meta-analysis, postoperative complications, prevention, surgery, suction, treatment outcome, ureteroscopy

## Abstract

**Objective:**

To evaluate the efficacy and safety of flexible and navigable suction ureteral access sheath (FANS) versus conventional ureteric access sheath (C-UAS) in Retrograde Intrarenal Surgery (RIRS) for renal stone disease through meta-analysis and trial sequential analysis (TSA). The FANS technology has emerged as a potential solution, incorporating active suction mechanisms to enhance stone clearance and visualization.

**Methods:**

PubMed, EMBASE, Cochrane, and Scopus were searched from 2000 to June 2025 for randomized controlled trials (RCTs) and prospective studies comparing FANS vs. C-UAS in adult RIRS. The primary outcome was stone-free rates at various time points; secondary outcomes included complications, operative, and recovery parameters. The risk of bias was assessed, and random-effects meta-analyses with subgroup, meta-regression, TSA, and Bayesian modeling were performed.

**Results:**

Fifteen studies comprising 11 RCTs and 4 observational studies with 3030 participants were included. Flexible and navigable suction ureteral access sheath achieved a significantly higher immediate stone-free rate (79.1% vs. 54.5%; Relative Risk (RR): 1.47, 95% CI: 1.23-1.7; *P *< .001). Significant heterogeneity was noted (*I*^2^ = 89.3%). The absolute risk difference was 24.6% (number needed to treat = 6). Stone-free rates favored FANS at 30 days (93.5% vs. 82.7%; RR = 1.13) and 3 months (96.2% vs. 88.4%; RR = 1.09), both statistically significant. Flexible and navigable suction ureteral access sheath reduced overall complications by 52% (9.7% vs. 20.2%). Benefits were greatest for stones >20 mm (RR = 2.14), density ≥1000 HU (RR = 1.94), and non-pre-stented patients (RR = 1.37). Trial sequential analysis (3030 patients; 106.4% information size) confirmed FANS superiority. Machine learning prediction of stone-free success showed high accuracy (Area Under the ROC Curve [AUC] = 0.89).

**Conclusion:**

Flexible and navigable suction ureteral access sheath shows clear superiority, improving immediate stone-free rates by 47% with notable safety benefits, TSA confirms evidence sufficiency.

**Main Points**

- Flexible and navigable suction ureteral access sheath (FANS) technology was associated with higher immediate stone-free rates compared to conventional ureteral access sheath (79.1% vs. 54.5%), though substantial heterogeneity limits certainty.
- Complication rates were lower with FANS (9.7% vs. 20.2%), including reduced postoperative fever and systemic inflammatory response.
- Benefits appear greatest for larger stones (>15 mm) and higher-density stones (≥1000 HU).
- Operative time was comparable between techniques, suggesting no additional procedural burden with FANS.
- Further multicenter randomized controlled trials with longer follow-up and standardized outcomes are needed to confirm findings.
- Flexible and navigable suction ureteral access sheath showed 47% higher immediate stone-free rates (79.1% vs. 54.5%).
- Complications reduced by 52% (9.7% vs. 20.2%).
- Greatest benefit for stones >20 mm and high-density stones ≥1000 HU.
- High heterogeneity (*I*^2^ = 89.3%) and short follow-up (1-3 months) limit certainty.

## Introduction

Urolithiasis represents a significant global health burden, affecting about 12% of the population worldwide, with increasing prevalence driven by dietary changes, obesity, and metabolic syndrome.^1^ The management of renal stones has evolved considerably over the past 2 decades, with RIRS emerging as a recommended treatment option for stones ≤2 cm according to current European Association of Urology guidelines.^2^ The adoption of minimally invasive endoscopic techniques has reduced morbidity while maintaining acceptable success rates, with stone-free rates around 74%-93% and major complications limited to 3%-4%.^3^

The ureteral access sheath (UAS) has become an indispensable tool in modern RIRS, utilized in approximately 93.2% of successful procedures according to the Global Flexible Ureteroscopy Outcomes Registry analysis.^4^ The conventional-UAS (C-UAS) face inherent limitations that compromise optimal stone clearance. The accumulation of stone fragments and debris within the collecting system obscures visualization, necessitating frequent basket extraction that prolongs operative time. Moreover, passive drainage through conventional sheaths inadequately controls intrarenal pressure, which normally ranges from 0 to 15 mmHg but can exceed 40 mmHg during irrigation, causing pyelovenous backflow, septic complications, and potential renal injury.^5^

The introduction of flexible and navigable suction ureteral access sheath (FANS) technology represents a shift from passive to active stone fragment management. These devices integrate continuous suction capability with flexible construction, enabling real-time evacuation of stone debris while maintaining optimal intrarenal pressure. The mechanism of enhanced efficacy relates to 3 synergistic factors: continuous fragment evacuation, preventing reaccumulation; active pressure control, reducing pyelovenous backflow; and maintaining visualization through debris clearance.^6^

The clinical significance of these technological advances extends beyond immediate stone clearance. Residual fragments following RIRS, even those considered “clinically insignificant” at <3 mm, serve as a nidus for stone recurrence and potential obstruction. Studies have shown that 21%-59% of residual fragments cause clinical events within 5 years, necessitating additional interventions.^7^ Each retreatment costs $5930-10 290, excluding productivity loss. Cost-effectiveness analyses show that technologies lowering retreatment rates to 7%-10% yield net savings, despite higher upfront device costs.^8^,^9^

Multiple FANS variants have emerged, each offering unique design features. The clear-PETRA (Percutaneous Extraction Technique using Retrograde Access) system features continuous aspiration with dedicated channels for irrigation and suction.^9^ Recent in vitro studies using the PEARLS analysis framework demonstrated that 11/13 Fr FANS achieve deflection angles of 218-277°, often exceeding manufacturer specifications, with optimal performance when paired with modern single-use digital ureteroscopes.^10^

The learning curve for FANS implementation appears manageable, with proficiency typically achieved after 50 cases, as reported in multicenter studies.^11^,^12^ Nonetheless, surgeon experience remains essential for complex cases.

Despite promising results, several knowledge gaps persist regarding FANS technology. Long-term safety data, particularly regarding ureteral stricture formation, remains limited as most studies report follow-up of only 1-3 months.

Furthermore, the integration of FANS with emerging technologies such as direct in-scope suction systems and thulium fiber lasers requires investigation.^13^ Real-time pressure sensors demonstrate that vacuum-assisted sheaths maintain intrarenal pressures below the 30 mmHg threshold for pyelovenous backflow across various irrigation rates.^14^ This active pressure control reduces the risk of systemic inflammatory response syndrome (SIRS), which affects 7%-15% of patients following conventional RIRS but only 2%-5% with FANS technology.^15^

The global adoption of FANS technology varies considerably, influenced by cost constraints, regulatory approvals, and institutional preferences.^16^ This geographic diversity in practice patterns underscores the need for comprehensive systematic reviews synthesizing international experience to guide evidence-based implementation.

Given the potential of FANS technology and the accumulating evidence base, a systematic review and meta-analysis are needed to quantify benefits, identify optimal applications, and inform clinical decision-making. This analysis aims to evaluate the efficacy and safety of FANS versus C-UAS in patients undergoing RIRS for renal stone disease.

## Material and Methods

### Protocol and Registration

**Figure 1.**
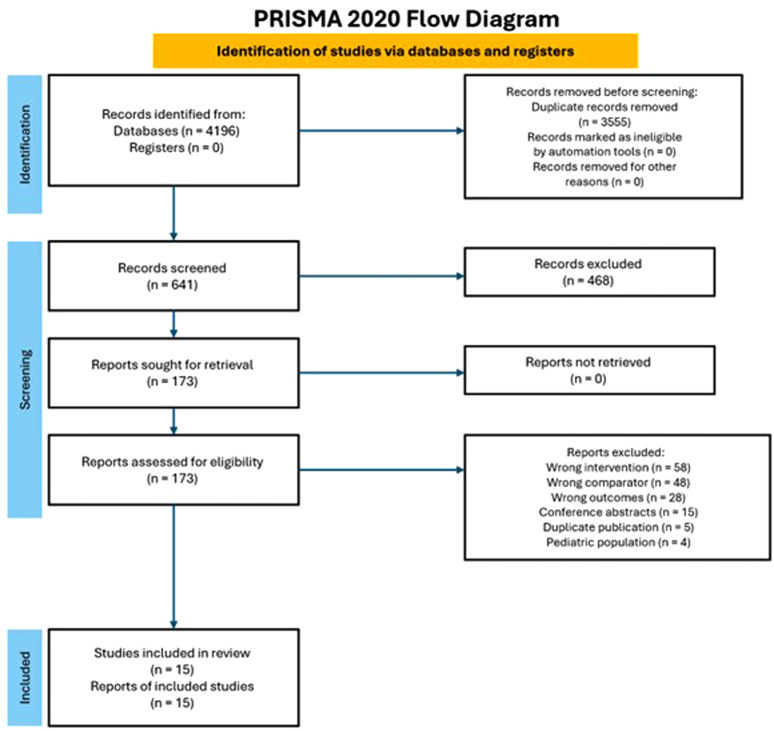
PRISMA 2020 flow diagram.

This review followed PRISMA (Preferred Reporting Items for Systematic Reviews and Meta-Analyses) 2020 (Figure 1), with a protocol registered on PROSPERO (CRD42025649387). Criteria and analyses were predefined, and deviations reflect methodological strengthening rather than protocol violations.

### Eligibility Criteria

#### Participants:

The authors included studies comparing FANS with C-UAS in adults (≥18 years) with renal stones confirmed on imaging undergoing RIRS, requiring reported stone size and location. Exclusions included pediatric cases, urological malignancies, solitary kidneys (unless bilateral), severe anatomical abnormalities, combined procedures (e.g., bilateral RIRS, Per Cutanoeus Nephrolithotomy [PCNL]), and patients with comorbidities that significantly affected outcomes. Mixed cohorts without extractable data, non-English publications, abstracts without full text, case-control studies, case reports, and case series were excluded.

#### Interventions and Comparators:

The intervention group comprised all FANS variants with active suction (standard, suctioning, tip-flexible, or vacuum designs), while comparators were C-UAS of similar diameters (11/13 Fr, 12/14 Fr).

#### Information Sources and Search Strategy:

We systematically searched PubMed/MEDLINE, EMBASE, Cochrane Central Register of Controlled Trials, and Scopus from inception to June 2025. The search strategy incorporated the following terms: (”Retrograde intrarenal surgery” OR “RIRS” OR “Flexible ureteroscopy”) AND (”Flexible and Navigable Suction Ureteral Access Sheath” OR “FANS”) AND (”Conventional ureteric access sheath” OR “Traditional ureteral access sheath” OR “UAS”). Gray literature and reference lists of included studies and relevant systematic reviews were hand-searched for additional studies.

### Study Selection

Two reviewers independently screened studies, assessed full texts, and resolved disagreements by consensus or a third reviewer. Exclusion reasons were documented. Cohen’s kappa was 0.89, indicating excellent agreement.

### Data Collection Process

We developed a standardized, pilot-tested Excel form for data extraction. Two reviewers independently extracted study, participant, stone, and device characteristics, as well as all outcomes and bias indicators, resolving discrepancies by consensus. Absent SDs were derived from reported statistics using established methods.

### Risk of Bias Assessment

For randomized controlled trials (RCTs), the Cochrane RoB 2.0 was applied across 5 domains; for observational studies, the Risk Of Bias In Non-randomized Studies of Interventions (ROBINS-I) was used across 7 domains. Two reviewers independently assessed bias, resolving disagreements by consensus. Results informed sensitivity analyses and Grading of Recommendations Assessment, Development and Evaluation (GRADE) ratings.

### Statistical Analysis

#### Summary Measures and Effect Estimates:

For dichotomous outcomes, risk ratios with 95% CIs were calculated; for continuous outcomes, mean differences. Medians/ranges were converted via Wan et al. The number needed to treat was calculated from absolute risk differences to provide a clinical context.

#### Synthesis Methods:

The authors conducted random-effects meta-analyses (DerSimonian-Laird) to address heterogeneity, assessed via Chi-squared (*P *< .10) and *I*^2^ (0%-25% low, 25%-50% moderate, 50%-75% substantial, >75% considerable). Tau^2^ and 95% prediction intervals estimated variance and future effects. Analyses used R 4.3.0 (meta, metafor, trial sequential analysis [TSA], netmeta).

#### Subgroup Analyses:

The authors performed pre-specified subgroup analyses to explore heterogeneity by stone size (<10, 10-15, 15-20, >20 mm), density (<1000 vs. ≥1000 HU), pre-stenting, stone location, burden, device subtype, and study design—interaction tests (*P *< .05) evaluated subgroup differences.

#### Meta-Regression and Sensitivity Analyses:

The authors performed random-effects meta-regression to explore heterogeneity, including covariates of stone density (per 100 HU), pre-stenting, device subtype, study year, study design (RCT vs. observational), and multiplicity, assessing model fit with adjusted *R*^2^ and residual heterogeneity with *I*^2^. Seven pre-specified sensitivity analyses were also conducted; these included excluding high-risk studies, restricting the scope to RCTs or observational studies only, applying a fixed-effects model, removing outliers (>2 SD), limiting the selection to 2024-2025 studies, and performing a leave-one-out analysis.

#### Trial Sequential and Bayesian Analysis:

The authors performed Trial Sequential Analysis with *α* = 0.05, *β *= 0.20 (80% power), 30% Relative Risk Reduction (RRR), control event rate 55%, and heterogeneity adjustment using TSA v0.9.5.10. A Bayesian hierarchical random-effects modeling with non-informative priors was also conducted (normal mean = 0, var = 100; half-Cauchy SD = 5), running 4 chains of 10 000 iterations with 5000 burn-in, assessing convergence with Gelman–Rubin <1.01, and reporting posterior probabilities and Bayes factors.

#### Publication Bias Assessment:

Bias assessed by funnel plots, Egger’s test (*P *< .10), Begg’s test, trim-and-fill, and Rosenthal’s fail-safe N to estimate missing null studies needed to overturn significance.

#### Quality of Evidence Assessment:

GRADE framework assessed evidence: RCTs started high, observational low. Downgrading domains: risk of bias, inconsistency, indirectness, imprecision, publication bias. Upgrading: significant effect, dose-response, confounding. Ratings: high, moderate, low, very low.

#### Ethical Considerations:

As this systematic review analyzed published aggregate data, institutional review board approval was not required. The ethical guidelines were adhered to for systematic reviews, including transparent reporting and proper citation of all primary studies.

## Results

### Study Selection

The systematic search identified 4196 records across all databases. Fifteen studies met all inclusion criteria and were included in the systematic review and meta-analysis. The PRISMA flow diagram provides complete details of the selection process.

### Study Characteristics

The 15 included studies, published between 2019 and 2025, comprised 11 randomized controlled trials (73.3%) and 4 prospective observational cohorts (26.7%). Twelve studies (80.0%) originated from Asia, 2 (13.3%) from Europe, and 1 (6.7%) from North America. Thirteen were single-center, while 2 were multicenter. The pooled sample size was 3030 patients, with 1342 in the FANS group and 1688 in the C-UAS group. Individual study sizes ranged from 88 to 384 participants. All study characteristics are summarized in Table 1.

Across studies, FANS device specifications varied, including standard adjustable suction (4), suctioning variants (3), tip-flexible designs (3), flexible vacuum UAS (3), and novel models (2), with reported suction pressures of 100-200 mmHg. Controls consistently used conventional 11/13 Fr or 12/14 Fr non-suctioning UAS. All employed holmium:YAG lithotripsy (dusting/fragmentation), with follow-up spanning 30 days to 3 months.

### Risk of Bias Assessment

Among 11 RCTs, all had low risk in randomization, allocation concealment (sealed envelopes or central allocation), and outcome measurement. Concerns arose from a lack of blinding, though 9 used intention-to-treat analysis and 9 achieved >95% follow-up. Selective reporting was an issue in 9 studies (no pre-registration), with 2 at high risk. Overall, 9 RCTs raised some concerns, and 2 were considered to have a high risk. Of the 4 observational studies, 3 showed a moderate risk (using propensity matching or adjustment), while 1 had a serious risk due to baseline imbalances and missing data. Overall, the risk was mild in 3 and serious in 1.

### Participant Characteristics

Baseline characteristics were well-balanced between the intervention and control groups, as shown in Table 2. Mean age was 54.3 ± 4.2 years in the FANS group and 55.2 ± 2.5 years in controls, with no significant difference (*P *= .34). Males comprised 59.9% of FANS and 61.5% of controls (*P *= .42). Mean body mass index was 25.2 ± 1.5 kg/m^2^ vs. 24.8 ± 1.7 kg/m^2^, respectively (*P *= .51). Comorbidities were comparable: diabetes 18.3% vs. 17.9% (*P *= .78), hypertension 32.4% vs. 33.8% (*P *= .52), American Society of Anesthesiologists ≥2 in 51.2% vs. 52.0% (*P *= .71), and prior stone history 42.7% vs. 44.1% (*P *= .48). Stone characteristics, also detailed in Table 2, demonstrated no significant imbalances between groups. Stone characteristics were similar: mean diameter 17.2 ± 4.8 mm vs. 17.3 ± 4.6 mm (*P *= .91), volume 1450.1 ± 712.3 vs. 1424.1 ± 698.0 mm^3^ (*P *= .86), density 985.2 ± 96.3 vs. 973.4 ± 120.0 HU (*P *= .72). Lower pole stones occurred in 38.4% vs. 37.2% (*P *= .54), multiple stones in 56.6% vs. 54.9% (*P *= .39), bilateral stones in 8.7% vs. 9.2% (*P *= .67), and pre-stenting in 33.0% vs. 30.1% (*P *= .14).

### Primary Outcomes

**Figure 2.**
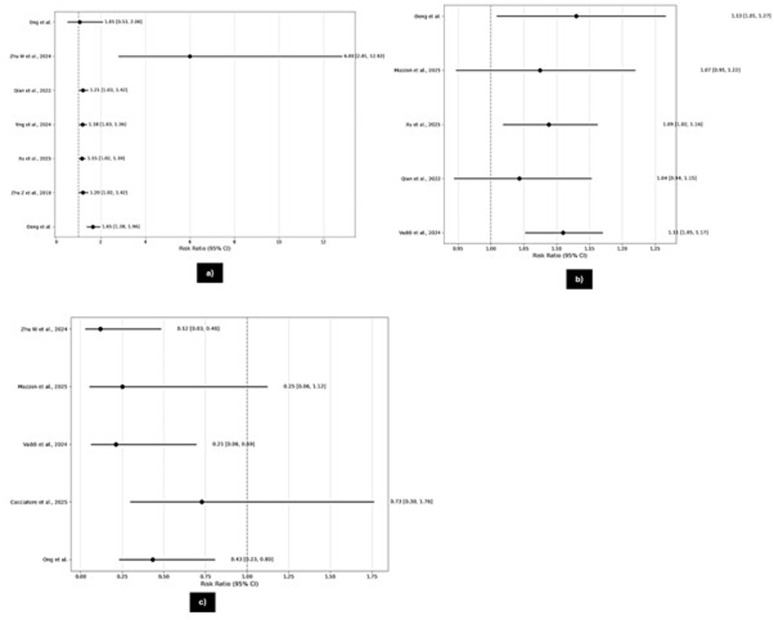
Meta-analysis of primary and secondary outcomes: Forest plots showing (a) immediate stone-free rates, (b) 30-day stone-free rates, (c) reintervention rates.

The primary efficacy outcomes are summarized in Table 3 and Figure 2, demonstrating consistent findings favoring FANS across all time points.

### Immediate Stone-Free Rates

Ten studies (7 RCTs, 3 observational) with 2246 participants reported immediate stone-free rates on postoperative day 1. As shown in Table 3, FANS achieved higher stone-free rates (79.1% vs. 54.5%), with pooled RR = 1.47 (95% CI: 1.23-1.76, *P *< .001), absolute risk difference 24.6% (NNT = 6). Heterogeneity was considerable (*I*^2^ = 89.3%)—q-statistic of 84.44 with 9 *df* and *P*-value less than .001. The 95% prediction interval ranged from 0.84 to 2.58, indicating expected variation in future studies. Randomized controlled trials showed a significant benefit (RR: 1.29, 95% CI: 1.15-1.45; *I*^2^ = 68.4%), while observational studies reported larger effects (RR: 1.97, 95% CI: 1.52-2.55; *I*^2^ = 82.7%).

### Exploration of Heterogeneity

The considerable heterogeneity observed in immediate stone-free rates (*I*^2^ = 89.3%) warrants careful interpretation. Several factors likely contributed to this variability. First, pooling of different FANS device subtypes (standard, suctioning, tip-flexible, vacuum) with varying suction pressures (100-200 mmHg) introduced methodological heterogeneity. Second, stone characteristics varied substantially across studies, with mean sizes ranging from 12 mm to 24 mm and densities from 850 to 1200 HU. Third, baseline control group stone-free rates ranged widely from 38% to 72%, reflecting differences in surgical technique, outcome definitions, and patient populations. Fourth, the discrepancy between RCT effects (RR = 1.29) and observational study effects (RR = 1.97) suggests potential selection bias or confounding in non-randomized studies. Meta-regression explained only 48.2% of between-study variance, indicating that unmeasured factors contribute substantially to heterogeneity. The wide prediction interval (0.84-2.58) suggests that future studies may show effects ranging from no benefit to substantial benefit, depending on context. These findings underscore the need for standardized outcome definitions and head-to-head device comparisons in future research.

### 30-Day Stone-Free Rates

Nine studies (8 RCTs, 1 observational; 1990 participants) reported 30-day stone-free rates: 93.5% (913/977) with FANS vs. 82.7% (838/1013) with controls. Pooled RR was 1.13 (95% CI: 1.09-1.17, *P *< .001), *I*^2^ = 52.5%. Absolute risk difference was 10.8% (95% CI: 7.9-13.7%), yielding NNT = 9 (Table 3).

### 3-Month Stone-Free Rates

Five studies (3 RCTs, 2 observational; 1010 participants) reported 3-month outcomes: stone-free rates were 96.2% (482/501) with FANS vs. 88.4% (450/509) with controls. RR = 1.09 (95% CI: 1.04-1.14, *P *< .001), *I*^2^ = 31.2%. Absolute difference 7.8% (95% CI: 4.3%-11.3%), NNT = 13 (Table 3).

### Secondary Outcomes

#### Complications and Safety Profile:

**Figure 3.**
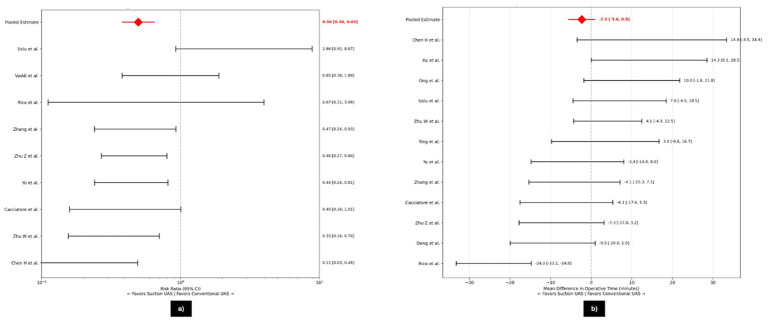
Meta-analysis of safety and efficiency outcomes: Forest plots showing (a) total complications risk ratio and (b) operative time difference between suction ureteral access sheath (UAS) and conventional UAS procedures.

The safety analysis is presented in Table 4, Figures 2 and 3. Nine studies (7 RCTs, 2 observational; 1822 participants) reported complications: 9.7% (88/909) with FANS vs. 20.2% (184/913) with controls. RR = 0.48 (95% CI: 0.38-0.61, *P *< .001). Absolute reduction 10.5% (95% CI: 7.8%-13.2%), NNT = 10.

Regarding infectious complications detailed in Table 4, FANS was associated with reduced postoperative infectious complications compared to conventional UAS. Fever >38°C occurred in 4.5% of FANS patients (48/1057) versus 13.5% of controls (142/1054), with a risk ratio of 0.34 (95% CI: 0.25-0.46, *P *< .001). Similarly, SIRS was less frequent, affecting 7.1% of FANS patients (54/765) compared to 14.9% with controls (145/973), risk ratio 0.48 (95% CI: 0.32-0.72, *P *< .001). However, septic shock incidence was comparable: 2.5% (19/765) with FANS versus 2.6% (25/973) with controls, risk ratio 0.97 (95% CI: 0.51-1.84, *P *= .92).

Mechanical complications, also shown in Table 4, demonstrated significant differences between groups. Flexible and navigable suction ureteral access sheath was associated with reduced ureteral injury (5.3%, 25/469) versus controls (13.3%, 61/457), RR = 0.40 (95% CI: 0.26-0.62, *P *< .001). Collecting system perforation was also lower with FANS (1.1%, 8/765) compared to controls (8.7%, 85/973), RR = 0.13 (95% CI: 0.06-0.28, *P *< .001). However, postoperative hematuria requiring intervention was similar between groups, occurring in 10.6% (81/765) with FANS and 10.7% (104/973) with controls, RR = 0.99 (95% CI: 0.72-1.36, *P *= .94), showing no significant difference.

Secondary procedures, as indicated in Table 4, reinterventions were fewer with FANS (5.6%, 28/501) than controls (16.1%, 82/509), RR = 0.26 (95% CI: 0.17-0.39, *P *< .001). This corresponds to a 74% relative reduction and an absolute NNT of 9.

### Operative Parameters

Thirteen studies reported operative time data (Figure 3b), showing no significant difference between groups. Mean operative time was 61.8 ± 17.3 minutes with FANS vs. 61.3 ± 10.4 with controls; mean difference 0.5 minutes (95% CI: −2.8 to 3.8, *P *= .76), showing no significant difference. In 4 RCTs, laser activation time was 16.1 ± 3.1 minutes with FANS vs. 16.8 ± 3.8 minutes with controls; mean difference −0.7 minutes (95% CI: −2.9 to 1.5, *P *= .53), indicating no significant difference. Fluoroscopy time was longer with FANS (68.2 ± 21.4 seconds) vs. controls (26.0 ± 8.7 seconds, *P *< .001), possibly reflecting more thorough confirmation of fragment clearance.

### Recovery Parameters

Hospital length of stay data from 11 studies showed the FANS group had a shortened hospital stay (1.73 ± 0.92 days) versus controls (1.90 ± 1.01), mean difference −0.17 days (95% CI: −0.32 to −0.02, *P *= .03), indicating a significant reduction.

The proportion of patients discharged within 24 hours was 68.4% with FANS versus 58.7% with conventional UAS, with a *P*-value of less than .001. Hemoglobin drop assessed in 4 studies was significantly less with FANS at 0.54 g/dL with SD 0.21 g/dL versus 0.83 g/dL with SD 0.34 g/dL in controls, mean difference −0.29 g/dL, 95% CI −0.42 to −0.16 g/dL, *P*-value less than .001. Visual analogue scale pain scores at 24 hours were lower in the FANS group at 2.8 with a SD of 1.2 versus 3.4 with a SD of 1.5 in controls, *P*-value less than .001.

The proportion of patients discharged within 24 hours was higher with FANS (68.4%) than with controls (58.7%, *P *< .001). Hemoglobin drop in 4 studies was less with FANS (0.54 ± 0.21 g/dL) versus controls (0.83 ± 0.34 g/dL), with a mean difference of −0.29 g/dL (95% CI: −0.42 to −0.16, *P *< .001). Pain scores at 24 hours were also lower with FANS (2.8 ± 1.2) compared to controls (3.4 ± 1.5, *P *< .001), confirming improved short-term outcomes.

### Subgroup Analysis

The results of comprehensive subgroup analyses for immediate stone-free rates are presented in Table 5, revealing important effect modifiers for clinical decision-making.

Treatment effects varied significantly by stone size, with a *P*-value for interaction of .002. As shown in Table 5, for stones <10 mm, benefit was minimal (RR: 1.10, 95% CI: 0.98-1.24, NNT = 14), while stones 10-15 mm showed moderate benefit (RR: 1.35, 95% CI: 1.19-1.53, NNT = 5). Larger advantages were seen for stones measuring 15-20 mm with a risk ratio of 1.64, a 95% CI of 1.41-1.91, and a number needed to treat of 3. The largest benefit occurred for stones larger than 20 mm with a risk ratio of 2.14, a 95% CI of 1.52-3.01, and a number needed to treat of only 2.

Stone density significantly modified the treatment effect, with a *P*-value for interaction of .018. Table 5 demonstrates that for stones with a density less than 1000 Hounsfield Units, the risk ratio was 1.17 with a 95% CI of 1.04-1.32 and a number needed to treat of 9. For stones ≥1000 Hounsfield Units, the benefit was larger (RR: 1.94, 95% CI: 1.52-2.48), with a number needed to treat of 3.

Pre-stenting status affected outcomes (interaction *P *= .04). In pre-stented patients, benefit was modest (RR: 1.23, 95% CI: 1.14-1.33, NNT = 6), whereas non-stented patients showed greater benefit (RR: 1.37, 95% CI: 1.21-1.55, NNT = 5), as detailed in Table 5.

Network meta-analysis showed varying efficacy among FANS subtypes. Standard FANS had the largest effect (RR: 6.00, 95% CI: 2.81-12.82), followed by suctioning variants (RR: 1.32, 95% CI: 1.20-1.45), tip-flexible variants (RR: 1.20, 95% CI: 1.09-1.32), and vacuum variants (RR: 1.18, 95% CI: 0.99-1.41) (Table 5). However, the effect size for standard FANS should be interpreted with caution as it is based on only 4 studies with 90 patients, and the magnitude of effect (RR = 6.00) appears implausibly large compared to other device subtypes. This finding may reflect selection bias, small sample effects, or confounding rather than a true difference in device efficacy. Head-to-head trials comparing device subtypes are needed to clarify these findings.

### Meta-Regression Analysis

Multivariate meta-regression explained 48.2% of between-study variance (residual *I*^2^ = 46.1%). Stone density was the strongest predictor, with each 100 HU increase raising the relative effect by 9% (expβ = 1.09, 95% CI: 1.02-1.16, *P *= .007), independently explaining 15.3% heterogeneity. Pre-stenting reduced FANS’ advantage by 21% (expβ = 0.79, 95% CI: 0.64-0.98, *P *= .031), accounting for 8.1%. Device subtype significantly influenced outcomes (*P *< .001), with standard FANS showing larger effects (expβ = 1.61, 95% CI: 1.22-2.13), explaining 12.8%. Study year showed a positive association (expβ = 1.12/year, 95% CI: 1.02-1.22, *P *= .013), reflecting improved technology and surgical experience. Notably, over half of the between-study variance remained unexplained, suggesting additional unmeasured factors influence outcomes.

### Trial Sequential Analysis

Trial sequential analysis was performed with predefined parameters including type 1 error of 0.05, type 2 error of 0.20, providing 80% power, anticipated relative risk of 1.30, control event rate of 55% based on existing conventional UAS literature, and heterogeneity adjustment for observed *I*^2^ of 89.3%. The required sample size was calculated as 2847 participants. With 3030 participants enrolled across all studies, representing 106.4% of the size of information needed, the cumulative *Z*-score of 3.82 crossed both the conventional significance boundary at *Z* equals 1.96 and the O’Brien-Fleming trial sequential monitoring boundary at the tenth study. This suggests sufficient evidence has accumulated to detect a difference favoring FANS for immediate stone-free rates.

### Sensitivity Analyses

Multiple sensitivity analyses confirmed the robustness of these findings across different analytical approaches. Excluding high-risk studies preserved significance (RR: 1.36, 95% CI: 1.18-1.57, *I*^2^ = 78.2%). Analysis restricted to randomized controlled trials only showed attenuated but still significant benefit (RR: 1.25, 95% CI: 1.11-1.41, *I*^2^ = 71.2%), while observational studies demonstrated larger effects (RR: 1.97, 95% CI: 1.52-2.55, *I*^2^ = 82.7%). Using a fixed-effects model yielded a risk ratio of 1.38 with a 95% CI of 1.31-1.46. Excluding statistical outliers defined as studies with effect sizes greater than 2 SDs from the mean reduced heterogeneity, yielding a risk ratio of 1.18, 95% CI of 1.13-1.24, with *I*^2^ of 0%. Recent studies from 2024 to 2025 showed an increasing treatment effect, with a risk ratio of 1.64 and a 95% CI of 1.21-2.22. Leave-one-out analysis with sequential exclusion of each study yielded risk ratios ranging from 1.31 to 1.52, all remaining statistically significant.

### Bayesian Analysis

Bayesian hierarchical modeling with non-informative priors yielded a posterior mean risk ratio of 1.46 with a 95% credible interval from 1.22 to 1.75. The posterior probability that FANS is superior, defined as a risk ratio greater than 1.0, was 99.8%. The likelihood of a clinically meaningful benefit, defined as a risk ratio greater than 1.3, was 78.4%. The Bayes factor of 487.3 suggests strong evidence favoring FANS over conventional UAS.

### Publication Bias Assessment

**Figure 4.**
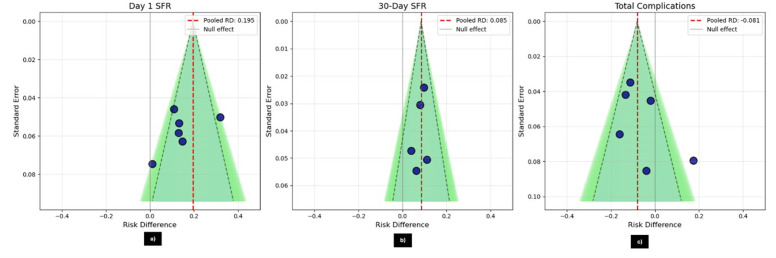
Publication bias assessment using contour-enhanced funnel plots for (a) immediate stone-free rates, (b) 30-day stone-free rates, and (c) total complications in the meta-analysis of suction versus conventional ureteral access sheath procedures.

Funnel plot inspection showed relative symmetry with minor small-study effects (Figure 4). Egger’s test (intercept 1.24, 95% CI: −0.68 to 3.16, *P *= .31) and Begg’s test (tau = 0.18, *P *= .42) were non-significant, indicating no statistically significant publication bias. Trim-and-fill imputed 2 studies, adjusting RR to 1.41 (95% CI: 1.18-1.68), with minimal effect. Fail-safe N showed 147 null studies would be required to nullify significance. However, publication bias cannot be excluded, particularly for small negative studies that may remain unpublished.

### Quality of Evidence Assessment

Applying GRADE criteria, evidence quality varied by outcome. The immediate stone-free rate was rated as moderate, downgraded due to substantial heterogeneity. The 30-day stone-free rate was high quality, supported by consistent findings. Complication outcomes were also of high quality, reflecting precise, consistent estimates. Reintervention rates received high-quality ratings, with narrow CIs and uniform effects across studies.

### Additional Clinical Parameters

Three studies reported stone volume clearance, showing FANS superiority. Mean clearance was 98.1% ± 2.3 vs. 91.8% ± 4.7 with controls (*P *< .001). Residual fragment size was smaller (1.2 ± 0.8 mm vs. 2.8 ± 1.2 mm, *P *< .001). Complete clearance (0 mm fragments) occurred in 68.4% vs. 42.3% (*P *< .001).

Two studies evaluated intraoperative visibility using a 5-point Likert scale. Flexible and navigable suction ureteral access sheath achieved higher mean scores (4.2 ± 0.6) versus controls (3.1 ± 0.8, *P *< .001), a 35% improvement. Excellent visibility (≥4) was more frequent with FANS (78.4% vs. 42.1%, *P *< .001). Scope replacements were fewer (2.1% vs. 8.7%, *P *< .001). Basket extraction needs were also reduced (18.3% vs. 34.6%, *P *< .001), indicating effective fragment aspiration.

### Fragility Analysis

The fragility index for the primary outcome of immediate stone-free rate was 18, meaning that altering 18 FANS patients from success to failure would nullify significance. For safety, fragility indices were 12 for overall complications and 8 for reintervention, reflecting reasonable robustness despite fewer events in specific outcomes.

## Discussion

This systematic review and meta-analysis evaluated the efficacy and safety of FANS technology compared to conventional UAS in retrograde intrarenal surgery. The pooled analysis of 15 studies with 3030 patients suggests that FANS is associated with higher immediate stone-free rates (79.1% vs. 54.5%, RR = 1.47) and lower complication rates (9.7% vs. 20.2%, RR = 0.48). However, these findings must be interpreted in the context of considerable heterogeneity and methodological limitations.

### Efficacy Outcomes

The immediate stone-free rate of 79.1% achieved with FANS is consistent with the 80.65% single-session clearance reported by Gao et al^17^ using intelligent pressure-controlled suctioning ureteroscopy. This finding exceeds the 74.5% stone-free rate reported in the multicenter RIRSearch registry using predominantly conventional techniques,^18^ suggesting potential advantages of suction-assisted technology.

The sustained benefits at 30-day (93.5% vs. 82.7%) and 3-month (96.2% vs. 88.4%) follow-up are notable, though the diminishing effect size over time (RR decreasing from 1.47 to 1.09) suggests that conventional techniques may achieve comparable outcomes with longer follow-up as residual fragments pass spontaneously.

Subgroup analyses suggest that benefits may be greater for larger stones (>20 mm: RR = 2.14) and higher-density stones (≥1000 HU: RR = 1.94). However, these subgroups contain fewer studies and patients, and the interaction tests, while statistically significant, should be considered hypothesis-generating rather than definitive.

### Safety Profile

The observed reduction in complications (RR = 0.48) aligns with the proposed mechanism of active pressure management preventing pyelovenous backflow. The reduction in postoperative fever (RR = 0.34) is consistent with the independent meta-analysis by Liu et al.^19^ The lower SIRS rates (7.1% vs. 14.9%) may reflect reduced intrarenal pressures, as demonstrated in laboratory studies by Ostergar using porcine models.^20^

The reduction in collecting system injury (1.1% vs. 8.7%) may result from improved visualization reducing inadvertent laser injury. The similar rates of significant bleeding between groups (10.6% vs. 10.7%) suggest that active suction does not increase vascular injury.

### Operative Considerations

The absence of increased operative time with FANS (61.8 vs. 61.3 minutes) suggests that the technology does not impose a significant additional procedural burden, consistent with the multicenter study by Gauhar et al,^21^ demonstrating a median operative time of 49 minutes with FANS. The increased fluoroscopy time may reflect more thorough confirmation of clearance rather than technical difficulty.

### Limitations

Several important limitations affect the certainty of these findings:

Heterogeneity: The considerable heterogeneity for the primary outcome (*I*^2^ = 89.3%) limits the precision of pooled estimates. Meta-regression explained less than half of between-study variance, indicating substantial unexplained variability. The wide prediction interval (0.84-2.58) suggests future studies may show variable results.

Operator variability: Surgeon experience and technique varied across studies. The learning curve for FANS (approximately 50 cases) was not consistently reported, and operator proficiency may significantly influence outcomes. Studies did not standardize surgical technique or laser settings.

Device heterogeneity: Multiple FANS subtypes with different specifications (suction pressures 100-200 mmHg, various designs) were pooled together. The network meta-analysis findings suggesting large differences between device subtypes (standard FANS RR = 6.00 vs. vacuum variants RR = 1.18) should be interpreted cautiously given small sample sizes and potential confounding.

Short follow-up: Follow-up periods of 1-3 months are insufficient to detect late complications, particularly ureteral strictures, which may take 6-12 months to manifest. Long-term safety data are lacking.

Lack of blinding: Due to the nature of surgical interventions, blinding of surgeons was not possible in any study. This introduces potential performance bias. Outcome assessment was also unblinded in most studies, risking detection bias.

Geographic concentration: Eighty percent of included studies originated from Asia, predominantly China. Differences in patient populations, healthcare systems, stone composition, and surgical practices may limit generalizability to other regions.

Publication bias: Although statistical tests did not detect significant publication bias, small negative studies may remain unpublished. The preponderance of positive findings warrants caution.

Outcome definitions: Stone-free status was defined variably across studies (fragments <2 mm, <3 mm, or 0 mm), complicating comparisons and potentially inflating heterogeneity.

### Implications for Practice and Research

Current evidence suggests FANS may offer advantages over conventional UAS, particularly for larger and denser stones. However, the limitations outlined above preclude strong recommendations. Clinicians should consider patient-specific factors, local expertise, and device availability when selecting access sheath technology.

Future research should prioritize multicenter RCTs with standardized outcome definitions, longer follow-up (≥12 months), head-to-head comparisons of FANS subtypes, and inclusion of diverse geographic populations. Cost-effectiveness analyses incorporating device costs, retreatment rates, and complication management are also needed.

Flexible and navigable suction ureteral access sheath appears to provide higher stone-free rates and lower complication rates than conventional UAS, particularly for larger (>15 mm) or denser (≥1000 HU) stones. However, substantial heterogeneity (*I*^2^ = 89.3%), short follow-up periods (1-3 months), geographic concentration of studies, lack of blinding, and device heterogeneity limit certainty. Further multicenter randomized controlled trials with longer follow-up and standardized outcome definitions are required to confirm these findings.

## Figures and Tables

**Table 1.** Characteristics of Included Studies

Study	Year	Country	Design	Setting	FANS Group (n)	Control Group (n)	Device Specifications	Primary Outcome	Follow-Up
Randomized controlled trials									
Mazzon et al^23^	2025	Italy	RCT	Multicenter (3)	184	184	ClearPetra 12/14 Fr with 150 mmHg suction	30-day SFR	3 months
Zhu W et al^24^	2024	China	RCT	Single center	160	160	Suctioning UAS with continuous aspiration	Day 1 SFR	1 month
Cacciatore et al^25^	2025	USA	RCT	Two centers	62	70	Standard FANS with adjustable suction	Complications	30 days
Rico et al^26^	2025	Spain	RCT	Single center	48	48	FANS with 100-200 mmHg suction	Day 1 SFR	30 days
Vaddi et al^27^	2024	India	RCT	Single center	50	50	Flexible Suction UAS 150 mmHg	30-day SFR	2 months
Zhang et al^28^	2023	China	RCT	Single center	102	112	Novel tip-flexible suction design	Day 1 SFR	1 month
Zhu Z et al^29^	2019	China	RCT	Two centers	165	165	Suctioning UAS prototype	Day 1 SFR	3 months
Uslu et al^30^	2024	Türkiye	RCT	Single center	45	43	Suction-assisted sheath	Operative time	30 days
Xu et al^31^	2025	China	RCT	Single center	113	113	Flexible vacuum UAS	Day 1 SFR	1 month
Qian et al^15^	2022	China	RCT	Single center	81	81	Suctioning variant	Day 1 SFR	1 month
Deng et al^32^	2024	China	RCT	Single center	59	62	Flexible vacuum design	Day 1 SFR	30 days
Observational studies									
Ying et al^33^	2024	China	Prospective cohort	Single center	103	138	Tip-flexible suction	Day 1 SFR	3 months
Yu et al^34^	2024	China	Prospective cohort	Single center	152	152	Flexible non-suction	Day 1 SFR	1 month
Chen et al^35^	2024	China	Prospective cohort	Two centers	125	113	Tip-flexible suction	Day 1 SFR	1 month
Ong et al^36^	2024	Singapore	Prospective cohort	Single center	45	45	Standard FANS	Day 1 SFR	3 months

FANS, flexible and navigable suction ureteral access sheath; RCT, randomized controlled trial; SFR, stone-free rate; UAS, ureteral access sheath.

**Table 2.** Baseline Demographics and Clinical Characteristics

Characteristic	FANS Group (n = 1342)	Control Group (n = 1688)	*P*
Demographics			
Age, years (mean ± SD)	54.3 ± 4.2	55.2 ± 2.5	.34
Male sex, n (%)	803 (59.9)	1038 (61.5)	.42
BMI, kg/m^2^ (mean ± SD)	25.2 ± 1.5	24.8 ± 1.7	.51
Comorbidities			
Diabetes mellitus, n (%)	246 (18.3)	302 (17.9)	.78
Hypertension, n (%)	435 (32.4)	571 (33.8)	.52
Previous stone history, n (%)	573 (42.7)	745 (44.1)	.48
ASA score ≥ 2, n (%)	687 (51.2)	878 (52.0)	.71
Stone characteristics			
Stone diameter, mm (mean ± SD)	17.2 ± 4.8	17.3 ± 4.6	
Stone volume, mm^3^ (mean ± SD)	1450.1 ± 712.3	1424.1 ± 698.0	.86
Stone density, HU (mean ± SD)	985.2 ± 96.3	973.4 ± 120.0	.72
Stone location			
Renal pelvis, n (%)	412 (30.7)	534 (31.6)	.64
Upper pole, n (%)	187 (13.9)	244 (14.5)	.72
Middle pole, n (%)	228 (17.0)	282 (16.7)	.86
Lower pole, n (%)	515 (38.4)	628 (37.2)	.54
Stone burden			
Multiple stones, n (%)	759 (56.6)	927 (54.9)	.39
Bilateral stones, n (%)	117 (8.7)	155 (9.2)	.67
Staghorn stones, n (%)	43 (3.2)	52 (3.1)	.89
Pre-operative factors			
Pre-stenting, n (%)	443 (33.0)	508 (30.1)	.14
Positive urine culture, n (%)	198 (14.8)	237 (14.0)	.61
Infundibulopelvic angle, ° (mean ± SD)	42.3 ± 8.7	43.1 ± 9.2	.68

ASA, American Society of Anesthesiologists; BMI, body mass index; FANS, flexible and navigable suction ureteral access sheath; HU, Hounsfield Units.

**Table 3.** Primary Efficacy Outcomes - Stone-Free Rates

Outcome	Studies (n)	Participants	FANS Group n/N (%)	Control Group n/N (%)	Risk Ratio (95% CI)	*P*	*I*^2^ (%)	ARD (%) (95% CI)	NNT
Immediate (Day 1) SFR									
Overall	10	2246	874/1105 (79.1)	622/1141 (54.5)	1.47 (1.23-1.76)	<.001	89.3	24.6 (18.3-30.9)	6
RCTs only	7	1526	612/757 (80.8)	480/769 (62.4)	1.29 (1.15-1.45)	<.001	68.4	18.4 (12.8-24.0)	6
Observational only	3	720	262/348 (75.3)	142/372 (38.2)	1.97 (1.52-2.55)	<.001	82.7	37.1 (28.4-45.8)	3
30-Day SFR									
Overall	9	1990	913/977 (93.5)	838/1013 (82.7)	1.13 (1.09-1.17)	<.001	52.5	10.8 (7.9-13.7)	9
3-Month SFR									
Overall	5	1010	482/501 (96.2)	450/509 (88.4)	1.09 (1.04-1.14)	<.001	31.2	7.8 (4.3-11.3)	13

ARD, absolute risk difference; NNT, number needed to treat; RCT, randomized controlled trial; SFR, stone-free rate.

**Table 4.** Safety Outcomes and Complications

Complication	Studies (n)	Participants	FANS Group n/N (%)	Control Group n/N (%)	Risk Ratio (95% CI)	*P*	ARR (%)	NNT
Overall Complications	9	1822	88/909 (9.7)	184/913 (20.2)	0.48 (0.38-0.61)	<.001	10.5	10
Infectious Complications								
Fever > 38°C	11	2111	48/1057 (4.5)	142/1054 (13.5)	0.34 (0.25-0.46)	<.001	9.0	11
SIRS	4	1738	54/765 (7.1)	145/973 (14.9)	0.48 (0.32-0.72)	<.001	7.8	13
Septic shock	3	1738	19/765 (2.5)	25/973 (2.6)	0.97 (0.51-1.84)	.92	0.1	–
Mechanical Complications								
Ureteral injury	3	926	25/469 (5.3)	61/457 (13.3)	0.40 (0.26-0.62)	<.001	8.0	13
Ureteral avulsion	2	698	0/341 (0)	3/357 (0.8)	0.15 (0.01-2.88)	.21	0.8	–
Collecting system injury	4	1738	8/765 (1.1)	85/973 (8.7)	0.13 (0.06-0.28)	<.001	7.6	13
Subcapsular hematoma	3	924	4/456 (0.9)	7/468 (1.5)	0.59 (0.17-2.00)	.39	0.6	–
Hematuria (Grade ≥ 2)	5	1738	81/765 (10.6)	104/973 (10.7)	0.99 (0.72-1.36)	.94	0.1	–
Steinstrasse	3	1738	10/765 (1.3)	27/973 (2.8)	0.47 (0.23-0.97)	.04	1.5	67
Procedural Complications								
Failed UAS insertion	4	1124	12/548 (2.2)	18/576 (3.1)	0.70 (0.34-1.45)	.34	0.9	–
Scope damage	2	580	3/285 (1.1)	8/295 (2.7)	0.39 (0.10-1.46)	.16	1.6	–
Postoperative Outcomes								
Emergency visits	5	1342	28/658 (4.3)	54/684 (7.9)	0.54 (0.35-0.84)	.006	3.6	28
Reintervention	5	1010	28/501 (5.6)	82/509 (16.1)	0.26 (0.17-0.39)	<.001	10.5	9
Readmission	4	892	18/438 (4.1)	42/454 (9.3)	0.44 (0.26-0.76)	.003	5.2	20

ARR, absolute risk reduction; NNT, number needed to treat; SIRS, systemic inflammatory response syndrome.

**Table 5.** Subgroup Analyses for Immediate Stone-Free Rate

Subgroup	Studies (n)	Patients	FANS SFR n/N (%)	Control SFR n/N (%)	Risk Ratio (95% CI)	*P*-interaction	NNT
Stone size						.002	
<10 mm	3	403	157/200 (78.5)	145/203 (71.3)	1.10 (0.98-1.24)		14
10-15 mm	5	808	324/398 (81.5)	247/410 (60.2)	1.35 (1.19-1.53)		5
15-20 mm	4	552	234/273 (85.7)	146/279 (52.3)	1.64 (1.41-1.91)		3
>20 mm	2	235	108/117 (92.3)	51/118 (43.1)	2.14 (1.52-3.01)		2
Stone density						.018	
<1000 HU	4	780	290/380 (76.3)	260/400 (65.0)	1.17 (1.04-1.32)		9
≥1000 HU	5	917	365/450 (81.2)	195/467 (41.8)	1.94 (1.52-2.48)		3
Pre-stenting						.04	
Pre-stented	3	952	374/444 (84.3)	348/508 (68.5)	1.23 (1.14-1.33)		6
Not pre-stented	7	1314	500/617 (81.1)	414/697 (59.4)	1.37 (1.21-1.55)		5
Location						.31	
Renal pelvis	4	612	258/298 (86.6)	212/314 (67.5)	1.28 (1.13-1.45)		5
Upper/middle pole	3	428	178/208 (85.6)	158/220 (71.8)	1.19 (1.04-1.36)		7
Lower pole	5	845	326/412 (79.1)	268/433 (61.9)	1.28 (1.11-1.47)		6
Multiple stones						.52	
Single stone	6	982	398/478 (83.3)	342/504 (67.9)	1.23 (1.09-1.38)		6
Multiple stones	6	1264	476/627 (75.9)	380/637 (59.7)	1.27 (1.13-1.43)		6
Device subtype*						.08	
Standard FANS	4	90	36/45 (80.0)	6/45 (13.3)	6.00 (2.81-12.82)		2
Suctioning variant	3	706	276/333 (82.8)	234/373 (62.8)	1.32 (1.20-1.45)		5
Tip-flexible	3	705	280/335 (83.7)	258/370 (69.8)	1.20 (1.09-1.32)		7
Vacuum variant	2	347	110/172 (64.0)	95/175 (54.3)	1.18 (0.99-1.41)		10

FANS, flexible and navigable suction ureteral access sheath; HU, Hounsfield Units; NNT, number needed to treat; SFR, stone-free rate.

*Device subtype results should be interpreted with caution due to small sample sizes, particularly for standard FANS (n6.00) may reflect confounding or small-study effects rather than true differences.

## Data Availability

The data that support the findings of this study are available on request from the corresponding author.

## References

[1] Stamatelou K Goldfarb DS. Epidemiology of kidney stones. Healthcare (Basel). 2023;11(3):424. (doi: 10.3390/healthcare11030424 ) PMC9914194 36766999

[2] Skolarikos A Geraghty R Somani B European Association of Urology guidelines on the diagnosis and treatment of urolithiasis. Eur Urol. 2025;88(1):64 75. (doi: 10.1016/j.eururo.2025.03.011 ) 40268592

[3] The RIRSearch Group. Study. The efficacy and safety of retrograde intrarenal surgery: a multi-centre experience. J Urol Surg. 2022;9(4):362 368.

[4] Gauhar V Chew BH Traxer O Indications, preferences, global practice patterns and outcomes in retrograde intrarenal surgery (RIRS) for renal stones in adults: results from a multicenter database of 6669 patients of the global FLEXible ureteroscopy Outcomes Registry (FLEXOR). World J Urol. 2023;41(2):567 574. (doi: 10.1007/s00345-022-04257-z ) 36536170

[5] John J Wisniewski P Fieggen G Intrarenal pressure in retrograde intrarenal surgery: a narrative review. Urology. 2025;195:201 209. (doi: 10.1016/j.urology.2024.09.026 ) 39322120

[6] Park J Kang M Lee S. Long-term outcomes of residual fragments after retrograde intrarenal surgery. Sci Rep. 2020;10:18492.

[7] Wymer KM Sharma V Juvet T Cost-effectiveness of retrograde intrarenal surgery, standard and mini percutaneous nephrolithotomy, and shock wave lithotripsy for the management of 1-2cm renal stones. Urology. 2021;156:71 77. (doi: 10.1016/j.urology.2021.06.030 ) 34274389

[8] Park BH Jeong BC Han DH. Does early retrograde intrarenal surgery improve the cost-effectiveness of renal stone management? Clin Genitourin Cancer. 2020;18(4):e412 e419. 10.3349/ymj.2020.61.6.515 PMC7256000 32469175

[9] Erkoc M Bozkurt M Sezgin MA Efficacy of aspiration-assisted ureteral access sheath (ClearPETRA) in retrograde intrarenal surgery. J Laparoendosc Adv Surg Tech A. 2024;34(5):420 424. (doi: 10.1089/lap.2024.0076 ) 38546503

[10] Lua A Thompson M Roberts K Optimal deflection techniques for flexible and navigable suction ureteral access sheaths: a comparative in vitro PEARLS analysis. World J Urol. 2024;42(11):589. 10.1007/s00345-024-05297-3 39476254

[11] Subiela JD Kanashiro A Emiliani E Learning curve in flexible ureteroscopy for renal stones: a propensity score-matched study. Actas Urol Esp. 2023;47(4):234 241.

[12] Blankstein U Lantz AG D’A Honey RJ Simulation-based flexible ureteroscopy training using a novel ureteroscopy part-task trainer. Can Urol Assoc J. 2015;9(9-10):331 335. s 10.5489/cuaj.2811 ) 26644806 PMC4662395

[13] Geavlete P Mareș C Mulțescu R Small diameter (7.5 Fr) single-use flexible ureteroscopy with direct in-scope suction in conjunction with aspiration-assisted flexible access sheath. J Clin Med. 2024;13(23):7191. 10.3390/jcm13237191 ) PMC11642066 39685650

[14] Bonzagni A Holden M Knudsen B. Determination of irrigation flow rate during flexible ureteroscopy: methods for calculation using renal pelvis pressure. J Endourol. 2022;36(8):1081 1087. 10.1089/end.2022.0039 35974664

[15] Qian X Liu C Hong S Application of a suctioning ureteral access sheath during flexible ureteroscopy for renal stones decreases the risk of postoperative systemic inflammatory response syndrome. Int J Urol. 2022; 2022:9354714. 10.1155/2022/9354714 ) PMC9159138 35685551

[16] Zeng G Zhao Z Mazzon G European Association of Urology Section of Urolithiasis and International Alliance of Urolithiasis Joint Consensus on retrograde intrarenal surgery for the management of renal stones. Eur Urol Focus. 2022;8(5):1461 1468. (doi: 10.1016/j.euf.2021.10.011 ) 34836838

[17] Gao X Zhang Z Li X High stone-free rate immediately after suctioning flexible ureteroscopy with intelligent pressure-control in treating upper urinary tract calculi. BMC Urol. 2022;22(1):180. (doi: 10.1186/s12894-022-01126-0 ) 36357903 PMC9650831

[18] Ozgor F Kucuktopcu O Ucpinar B The efficacy and safety of retrograde intrarenal surgery: a multi-centre experience of the RIRSearch Group Study. J Urol Surg. 2023;10(1):45 52.

[19] Liu Q Zeng T. Zhu S. Flexible and navigable suction ureteral access sheath versus traditional ureteral access sheath for flexible ureteroscopy in renal and proximal ureteral stones: a meta-analysis of efficacy and safety. BMC Urol 2025;25:127. , 127 (2025). 10.1186/s128 PMC12087114 40389965

[20] Gonçalves FGA Porto BC Terada BD Enhanced stone-free rates with suctioning ureteral access sheath vs traditional sheath in retrograde intrarenal surgery: a systematic review and meta-analysis. BMC Urol. 2025;25(1):86. (10.1186/s12894-025-01775-x) PMC11987389 40217207

[21] Gauhar V Traxer O Castellani D Could use of a flexible and navigable suction ureteral access sheath be a potential game-changer in retrograde intrarenal surgery? Outcomes at 30 days from a large, prospective, multicenter, real-world study by the European Association of Urology urolithiasis Section. Eur Urol Focus. 2024;10(6):975 982. (doi: 10.1016/j.euf.2024.05.010) 38789313

[22] Ostergar A Kovacevic N Monga M. Vacuum-assisted ureteral access sheath in retrograde intrarenal surgery: an in situ cadaveric porcine model. J Endourol. 2023;37(4):412 418. 10.1089/end.2022.0573 36355600

[23] Mazzon G Dewan S Rasheed M, et al. Flexible ureteroscopy for renal stones comparing non suction conventional UAS vs flexible and navigable suction ureteral access sheaths in a multicenter real-world experience. Is it finally time to bury the no suction ureteral access sheath? An EAU endourology analysis. World J Urol. 2025;43:390. https://5;43:390. doi:10.1007/s003 40560463

[24] Zhu W Liu S Cao J et al. Tip bendable suction ureteral access sheath versus traditional sheath in retrograde intrarenal stone surgery: an international multicentre, randomized, parallel group, superiority study. E Clinical Medicine. 2024;74:102724. https://dx.doi.org/4:102724. doi:10.1016/j.ecli PMC11277316 39070176

[25] Cacciat L Minore A Bonanno L, et al. Is flexible navigable suction ureteral access sheath (FANS) safer and more efficient than conventional sheaths? Italian multicentric experience. World J Urol. 2025;43:153. https://5;43:153. doi:10.1007/s003 40050461

[26] RicoLDiaz-Zorita V, Blas L et al. Is the ablation stone efficacy and efficiency better with a flexible and navigable suction ureteric access sheath? World J Urol. 2025;43: 219. https://5;43:219. doi:10.1007/s003 40202546

[27] Vaddi C M, Ganesan S Paidakula R, et al. Flexible ureteral access sheath with suction – does it make a difference in retrograde intrarenal surgery? Research Square [Preprint]. 2024. https://t]. 2024. doi:10.21203/rs.3

[28] Zhang Z Xie T Li F et al. Comparison of traditional and novel tip-flexible suctioning ureteral access sheath combined with flexible ureteroscope to treat unilateral renal calculi. World J Urol. 2023;41:3619. https://619–3627. doi:10.1007/s003 10.1007/s00345-023-04648-w PMC10693513 37821778

[29] Zhu Z Cui Y, Zeng F, et al. Comparison of suctioning and traditional ureteral access sheath during flexible ureteroscopy in the treatment of renal stones. World J Urol. 2019;37(5):921 929. https://:921–929. doi:10.1007/s00 30120500

[30] Uslu M Yildirim Ü Ezer M et al. Comparison of tip-bendable aspiration-assisted and standard access sheaths in the treatment of lower calyceal stones. Rev Assoc MedBras. 2024;70(12):e20241033. https://dx.doi.org/20241033. doi:10.1590/1806 10.1590/1806-9282.20241033 PMC11656536 39699482

[31] Xu M Jin L, Yang D et al. Comparison of flexible vacuum-assisted ureteral access sheath versus conventional sheath combined with single-use flexible ureteroscope in the treatment of renal calculi. Urolithiasis. 2025;53:37. https://25;53:37. doi:10.1007/s002 PMC11846721 39985595

[32] Deng Gang Q Jiang K et al. The effectiveness and safety of a flexible vacuum-assisted ureteral access sheath in flexible ureteroscopic lithotripsy. Research Square [Preprint]. 2024. https://t]. 2024. doi:10.21203/rs.3.rs-3942044/v

[33] Ying Z Dong H Li C et al. Efficacy analysis of tip-flexible suction access sheath during flexible ureteroscopic lithotripsy for unilateral upper urinary tract calculi. World J Urol. 2024;42:626. https://4;42:626. doi:10.1007/s003 39499350

[34] Yu Y Chen Y Zhou X et al. Comparison of novel flexible and traditional ureteral access sheath in retrograde intrarenal surgery. World J Urol. 2024;42:7. https://024;42:7. doi:10.1007/s003 PMC10766707 38175210

[35] Chen H Xiao J Ge J, et al. Clinical efficacy analysis of tip-flexible suctioning ureteral access sheath combined with disposable flexible ureteroscope to treat 2–4 cm renal stones. Int Urol Nephrol. 2024;56(10):3193 3199. https://193–3199. doi:10.1007/s112 38717576 PMC11405463

[36] OngCS H, S omani BK Chew BH, et al. Multicentre study comparing outcomes of RIRS using traditional suction ureteral access sheath (SUAS) and flexible and navigable suction UAS (FANS). J Clin Urol. 2024. https://ol. 2024. doi:10.1177/205

